# Why HIV Virions Have Low Numbers of Envelope Spikes: Implications for Vaccine Development

**DOI:** 10.1371/journal.ppat.1004254

**Published:** 2014-08-07

**Authors:** John Schiller, Bryce Chackerian

**Affiliations:** 1 Laboratory of Cellular Oncology, National Cancer Institute, National Institutes of Health, Bethesda, Maryland, United States of America; 2 Department of Molecular Genetics and Microbiology, University of New Mexico School of Medicine, Albuquerque, New Mexico, United States of America; University of Florida, United States of America

## Introduction

**Figure 1:**
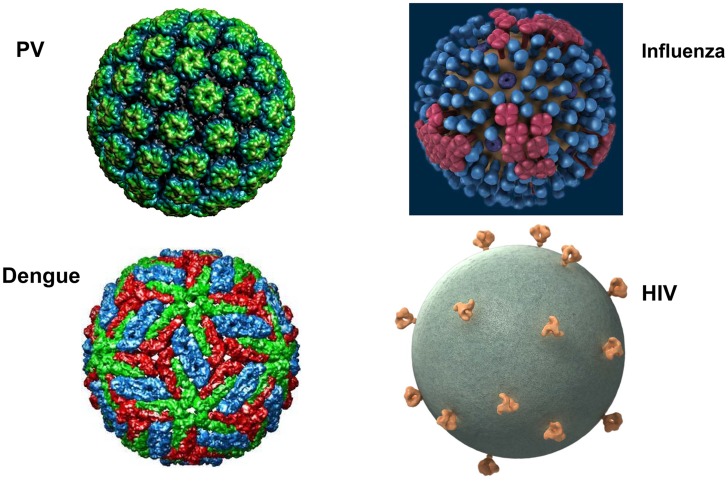
What's different about HIV virions? Surface projections of bovine papillomavirus (BPV), HIV, dengue virus, and influenza virus are shown (not to scale). The images of papillomavirus (PV) and dengue virus were obtained from the Viper database (PMID: 18981051). The image of influenza virus is courtesy of cdc.gov. The HIV image was generously provided by Sriram Subramaniam, National Cancer Institute.

The major structural proteins of most viruses, including both naked icosahedral and enveloped types, are present in a dense array on the virion surface. This pattern has likely evolved to promote structural integrity, maximize cell binding and entry, and minimize genome size. HIV and related simian lentiviruses are unusual in having a low density of envelope protein spikes on their surfaces (Figure 1) [1]. Why has HIV evolved this exceptional virion structure? We believe that studies of human papillomavirus (HPV) virus-like particles (VLPs), which are the basis of the highly successful HPV vaccine, may provide the conceptual underpinning for answering this question and key insights for developing effective HIV prophylactic vaccines.

## Why Does HIV Have So Few Virion Spikes?

HIV is notably inefficient at transmission—it has been estimated that 200–2,000 encounters are required per heterosexual transmission event [2]. In large part this may be due to the low number (estimated to be 14, on average) of envelope spikes per virion, especially since increasing the number of envelope spikes per virion increases infectivity, at least in an in vitro simian immunodeficiency virus (SIV) model [1], [3]. It seems unlikely that HIV evolved a low number of envelope spikes to limit its transmission efficiency. Low density of env spikes may have evolved, in part, because it prevents bivalent binding of immunoglobulin G (IgG) antibodies to the virion, thus reducing antibody avidity and impeding neutralization [4]. However, we think that an alternative explanation for the selection for low numbers of envelope spikes is that this feature retards the induction of a broad spectrum antibody response.

## Why Is It Important to Understand How a Virus Counteracts the Host's Antibody Response?

To be successful, mammalian viruses must overcome the host's humoral immune response. One strategy is to essentially outrun immunity by rapidly replicating to produce sufficient virions for transmission before neutralizing antibody can be generated. Alternatively, viruses that establish persistent infections must evade or delay induction of virus neutralizing antibodies so that infectious virions can be shed for extended periods [5]. The evasion mechanisms used by persisting viruses to circumvent humoral immunity vary depending upon the specifics of the viral life cycle. Understanding the major mechanisms of humoral immune evasion for a virus can provide key insights into development of effective antibody-mediated prophylactic vaccines against that virus, as recently demonstrated for HPVs. HPVs only produce virions in terminally differentiated layers of a stratified epithelium and release their virions to the environment during sloughing of the surface cells. As a consequence, antibody responses to natural HPV infection are weak [6]. However, by evolving an escape mechanism that relies on ignorance of the virions by the humoral immune system, HPVs have not evolved strategies to evade induction of potent neutralizing antibody responses if the virions are exposed to the systemic immune system, as is readily accomplished by standard parenteral injection. Thus, it is not surprising, at least in retrospect, that intramuscular injection of HPV L1 VLPs (which mimic the outer surface of the authentic virion) induces a virion antibody response that is highly protective against HPV infection and associated neoplasia [7].

## Why Do HPV Vaccines Induce Such Potent Neutralizing Antibody Responses?

Vaccination with HPV VLPs induces exceptionally strong antibody responses in humans. Even a single dose consistently induces neutralizing antibody titers that plateau well above the levels induced by natural infection, and they remain stable for years [8]. This type of humoral response, which is not seen with simple subunit vaccines, is thought to depend on the high-density repetitive display of the neutralizing epitopes on the VLP surface. Multivalent and dense (50–100 Å) spacing of surface determinants is a common feature of many microbial surfaces, including virions (Figure 1) and bacterial pili, and this pattern is a key determinant for recognition of an antigen as foreign by the humoral immune system [9]. This spacing of epitopes is rarely found on mammalian body surfaces routinely exposed to the systemic immune system. Clustering of B cell receptors by antigens with this spacing presumably sends exceptionally strong downstream activation and survival signals to the B cells, promoting high-titer and durable antibody responses. Remarkably, recognition of this epitope pattern is dominant over the mechanisms that normally promote B cell tolerance to self. For example, we found that display of tumor necrosis factor alpha (TNFα), a normally tolerogenic self-antigen, in a high-density array on an HPV VLP leads to long-lasting IgG responses against TNFα that are essentially equivalent to those against the foreign epitopes on the VLP [10]. The key determinant for breaking tolerance is the density of the epitope spacing [11]. Neither strong foreign T helper epitopes, high doses, nor potent adjuvants can substitute for high-density display for breaking self-tolerance. Vaccination with self-antigen displayed on VLPs, but not the same antigen in a low-valency form, can even reactivate self-specific anergic B cells [12]. The concept of virus-like display for breaking B cell tolerance to self-antigens has been well validated in clinical trials targeting several chronic disease targets [13].

## Why Has the Mammalian Immune System Evolved to Tolerate the Potential for Autoantibody Induction by Virus-Like Display?

**Figure 2:**
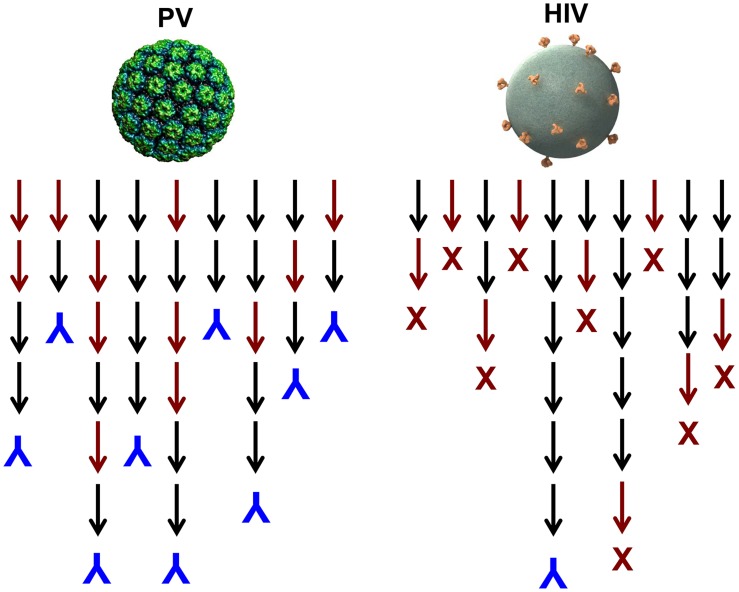
Model for the induction of neutralizing antibodies by high-density versus low-density epitope display. Black arrows represent antibody species during lineage evolution that are not reactive with self; red arrows represent species that are reactive with self. Inverted blue Ys denote B cell lineages that result in production of a virion binding antibody; red Xs denote deletion or tolerization of a self-reactive B cell lineage.

One could imagine induction of autoantibodies occurring, for instance, if virions become coated by their cell surface receptor during lytic infections. It is possible that virus-like display of self-antigens occurs so rarely that there has been no evolutionary pressure against it. However, we postulate that there has been positive selection for this potentially detrimental outcome because of the overwhelming importance of inducing a rapid neutralizing antibody response to lytic viruses. A majority of newly produced naive B cells are autoreactive [14] and functionally anergic [15], and many more B cells are likely to go through autoreactive intermediates during the process of somatic hypermutation that leads to affinity maturation. It follows that generating high-affinity neutralizing antibodies against high-density determinants on virion surfaces should be able to proceed along many immunoglobulin gene lineages because they could involve self-reactive progenitors and intermediates, thus ensuring their rapid and consistent appearance in a genetically diverse population (Figure 2). This conjecture is consistent with the observation that HPV VLPs induce essentially 100% seroconversion rates in humans, even after a single dose [8].

## Why Are Neutralizing Antibody Responses to HIV Infection Delayed?

In contrast to HPV VLP vaccination, it is well documented that neutralizing antibodies are exceptionally slow to develop during HIV infection, and nearly all broadly neutralizing antibodies invariably have undergone a large number of hypersomatic mutations [16]. Why is this the case for HIV and presumably not for most other viruses? Although the well-documented mechanisms involving glycan shields and conformational masking of key conserved functional sites likely contribute [17], we believe that the answer may primarily lie in the exceptionally low number of envelope spikes on the virion surface. In experimental systems, it has been shown that virus-like display of self-antigens can only overcome B cell tolerance when the mean epitope spacing is less than 100–150 Å [11]. The average spacing of HIV spikes has been estimated to be approximately 230 Å [1]. Therefore, in contrast to most other viruses, HIV neutralizing antibodies will likely have a much more constrained number of immunoglobulin gene developmental pathways because they cannot readily involve self-reactive intermediates (Figure 2). The low density of virion epitopes also raises the potential for HIV to evolve critical envelope determinates that are partially cross-reactive with self, so that mechanisms of self-tolerance can hinder induction of antibodies to them. Supporting this hypothesis is a recent study identifying a self-antigen that displays the epitope recognized by 2F5, a broadly neutralizing gp41 monoclonal antibody. Polypeptides bearing the epitope recognized by 2F5 are poorly immunogenic across species in which this epitope is conserved, but high-titer antibodies are readily induced in opossums, which naturally carry a variant peptide in the self-antigen that is not recognized by the antibody [18]. This immune evasion mechanism would not be available to viruses with high-density display of their critical virion determinants since the cross-reactive B cells would not be tolerized. This conjecture provides an explanation for the observation that most of the rare broadly neutralizing HIV antibodies isolated to date have an exceptionally high degree of self cross-reactivity and polyreactivity [19]. Given the need to develop through intermediates that are not strongly cross-reactive and an evolutionary drive to mimic self, it should not be surprising that broadly neutralizing HIV antibodies have complex lineages involving a high number of somatic mutations and develop late, if at all, in HIV-infected individuals.

## How Can Insights into HIV's Mechanism for Retarding Induction of Neutralizing Antibodies Be Translated into an Effective HIV Prophylactic Vaccine?

Recently, there have been suggestions for developing HIV prophylactic vaccines based on an in-depth understanding of the specific set of complex intermediates involved in the generation of broadly neutralizing antibodies during natural HIV infection [16], [19]. The observations outlined above suggest an alternative approach, bypassing the need to go through these specific intermediates by generating a virus-like display vaccine that could potentially generate broadly neutralizing antibodies through a larger set of lineages involving self-reactive intermediates. The key would be to generate high-density virus-like display of the HIV antigen. We favor display of conformationally correct forms of trimeric gp160, gp120, or gp41 over short peptides or mimotopes because it would be more difficult for the virus to escape a neutralizing antibody response encompassing multiple epitopes. Display of these large, complex antigens at sufficient density to break B cell self-tolerance may not be easy to achieve. In our opinion, this goal should be a major focus of HIV prophylactic vaccine development. Since HIV env epitopes may be more self cross-reactive than the capsid proteins of most viruses, it will be critical to carefully evaluate the possibility that pathogenic autoantibodies are generated by the vaccines.

It is important to note that not all “virus-like” particles are equally immunogenic in humans. For example, in contrast to HPV VLPs, the hepatitis B surface antigen particles that are the basis of all current hepatitis B virus (HBV) vaccines generate detectable antibodies in only a minority of subjects after one dose and titers do not routinely stabilize, even after three doses [20]. To inform design of prophylactic vaccines for HIV, it seems worthwhile to determine the key differences that account for the variability in the intrinsic immunogenicity of various VLP platforms and to evaluate their ability to break B cell tolerance to displayed self-antigens.

In summary, we postulate that HIV has evolved an exceptionally low number of envelope spikes so that effective neutralizing antibodies can't be generated through self-reactive intermediates and therefore may not have evolved effective defenses against vaccines that can. In trading low efficiency of transmission for delayed induction of an effective neutralizing antibody response, the virus may have created an Achilles heel that might be exploited by a vaccine that relies on high-density virus-like display.
